# Supplementary material for: A fluorescent perilipin 2 knock-in mouse model reveals a high abundance of lipid droplets in the developing and adult brain
Source: Nat Commun. 2024 Jun 28;15:5489. doi: 10.1038/s41467-024-49449-w (PMC11213871; doi:10.1038/s41467-024-49449-w)
Supplement: Supplementary file 1 — Supplementary Information [file 41467_2024_49449_MOESM1_ESM.pdf]

Supplementary information

A fluorescent perilipin 2 knock-in mouse model reveals a high abundance of lipid droplets in the developing and adult brain

Sofia Madsen[1], Ana C. Delgado[2], Christelle Cadilhac[3], Vanille Maillard[1], Fabrice Battiston[1], Carla Marie Igelbüscher[1], Simon De Neck[4], Elia Magrinelli[5], Denis Jabaudon[3], Ludovic Telley[5], Fiona Doetsch[2] and Marlen Knobloch[1*]

[1] Department of Biomedical Sciences, University of Lausanne, Lausanne, Switzerland. [2] Biozentrum, University of Basel, Basel, Switzerland. [3] Department of Basic Neurosciences, University of Geneva, Geneva Switzerland. [4] Institute of Veterinary Pathology, University of Zurich, Zurich, Switzerland.
[5] Department of Fundamental Neurosciences, University of Lausanne, Lausanne, Switzerland.

This file contains 8 supplementary figures and figure legends

Supplementary Figure 1

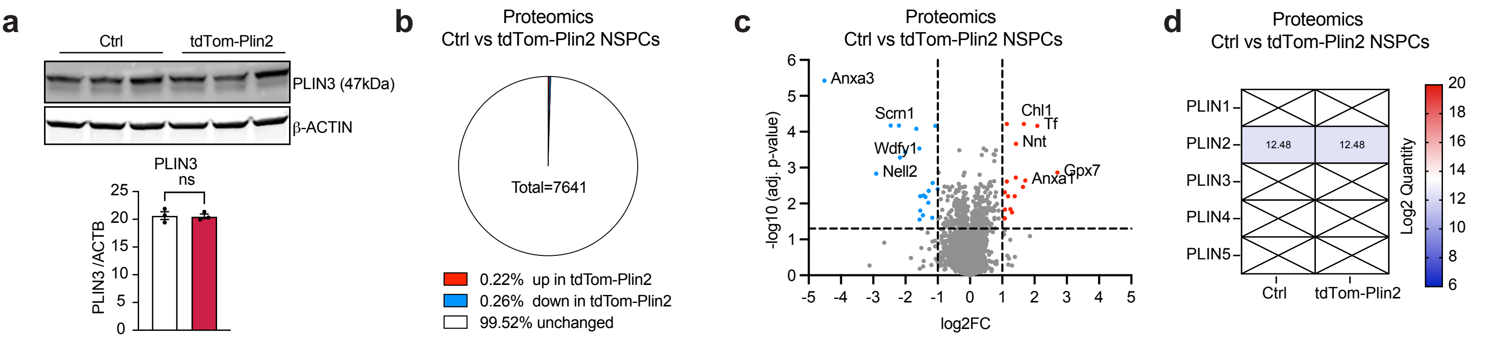

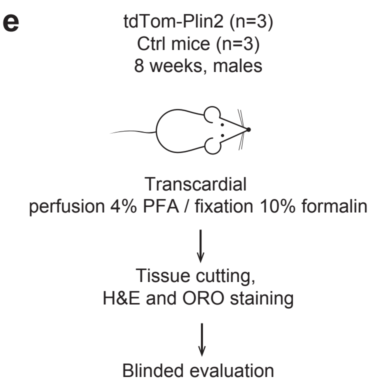

f

Tissue (H&E sections)	Observation
Adrenal gland	no difference
Bone (vertebra)	no difference
Bone and joint (femoro-tibial joint)	no difference
Bone and joint (sternum)	no difference
Bone marr ow (femur)	no difference
Bone marrow (sternum)	no difference
Bone marrow (tibia)	no difference
Bone marrow (vertebra)	no difference
Brain	no difference
Epididymis	no difference
Gall bladder	no difference
Harderian gland	no difference
Heart	no difference
Intestine, Large	no difference
Intestine, Small	no difference
Kidney	no difference
Larynx	no difference
Liver	no difference
Lung	no difference
Lymph node, Mandibular	no difference
Lymph node, Mesenteric	no difference
Ocular globe	no difference
Oesophagus	no difference
Pancreas	no difference
Preputial gland	no difference
Prostate	no difference
Salivary gland, Parotid	no difference
Salivary gland, Sublingual	no difference
Salivary gland, Submandibular	no difference
Seminal vesicle	no difference
Skeletal muscle (Diaphragm)	no difference
Skeletal muscle (thigh muscle)	no difference
Skin (haired)	no difference
Spinal cord, Lumbar/Cauda equina	no difference
Spinal cord, Thoracic	no difference
Spleen	no difference
Stomach	no difference
Testes	no difference
Thymus	no difference
Tongue	no difference

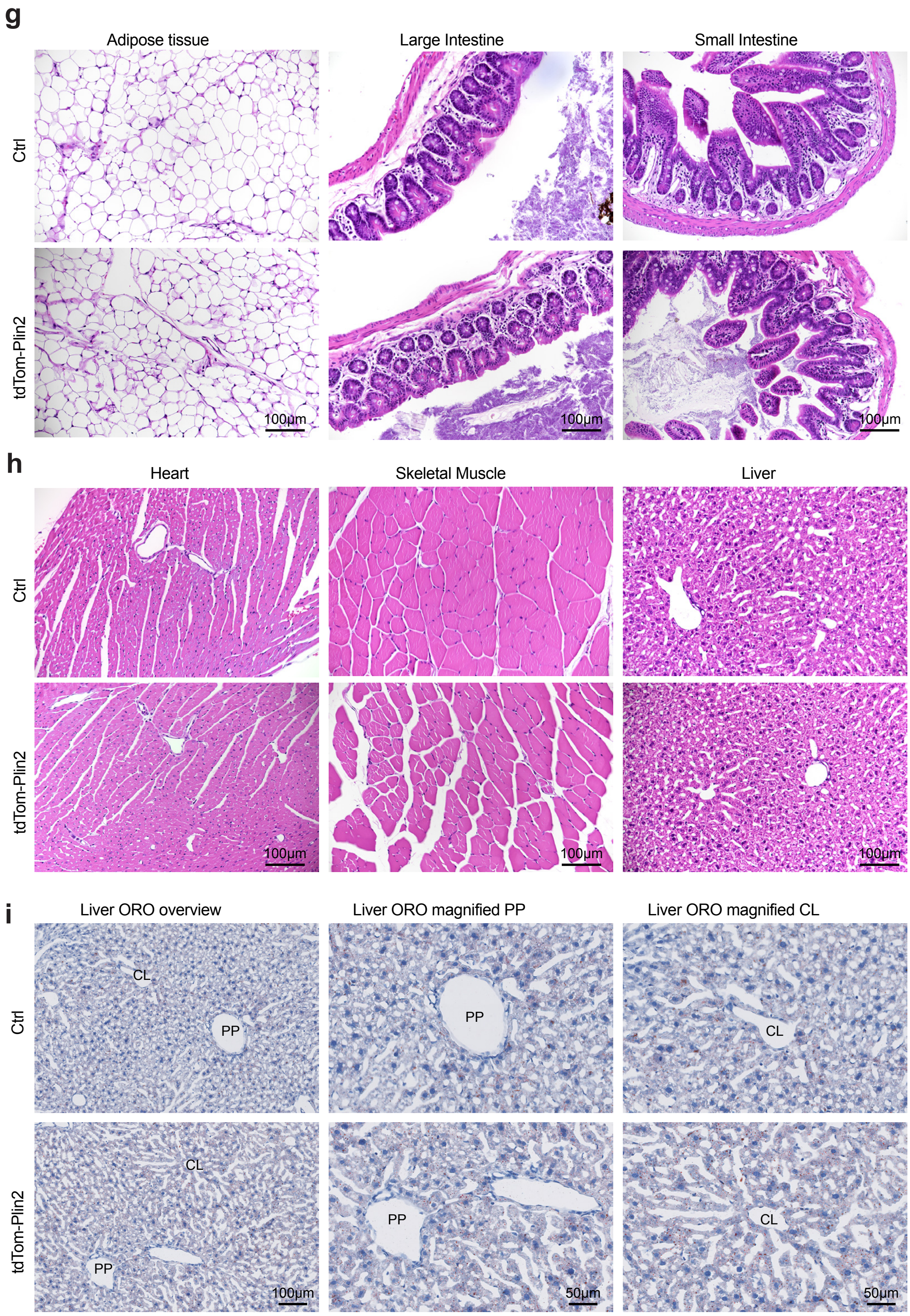

Supplementary Figure 1. Histopathological validation of the tdTom-Plin2 reporter mouse and further validation in tdTom-Plin2 NSPCs, related to figure 1. **a)** Western blot analysis shows that PLIN3 is not altered in NSPCs from the tdTom-Plin2 mice compared to Ctrl NSPCs (n=3 samples per condition, Blots repeated 3 times with similar outcome, mean +/- SEM, uncropped blots in Suppl. Figure 8). **b and c)** Proteomics analysis of tdTom-Plin2 NSPCs and Ctrl NSPCs shows that 99.52% of the proteins are unchanged. The changed proteins are not related to lipid metabolism (n=4 samples per condition). **d)** Only PLIN2 is sufficiently present to be detected by proteomics, underlining its importance for NSPCs. PLIN2 levels are the same in Ctrl and tdTom-Plin2 NSCPs. Shown is a heatmap of the median values of the log2 quantity (n=4 samples per condition). **e)** Schematic representation of the histological evaluation (n=3 mice per genotype), performed by a certified pathologist. **f)** Overview of the organs and tissues analyzed. “No difference” indicates that tissue from the two genotypes could not be distinguished. **g and h)** Representative images of hematoxylin and eosin (H&E) stained sections of organs known to contain LDs, such as adipose tissue, intestine, heart, muscle, and liver. The histological evaluation did not reveal any differences between Ctrl and tdTom-Plin2 mice. **i)** Overview and high magnification images of Oil red O (ORO) stained liver sections. Periportal (PP) and centrilobular (CL) regions are shown.

Supplementary Figure 2

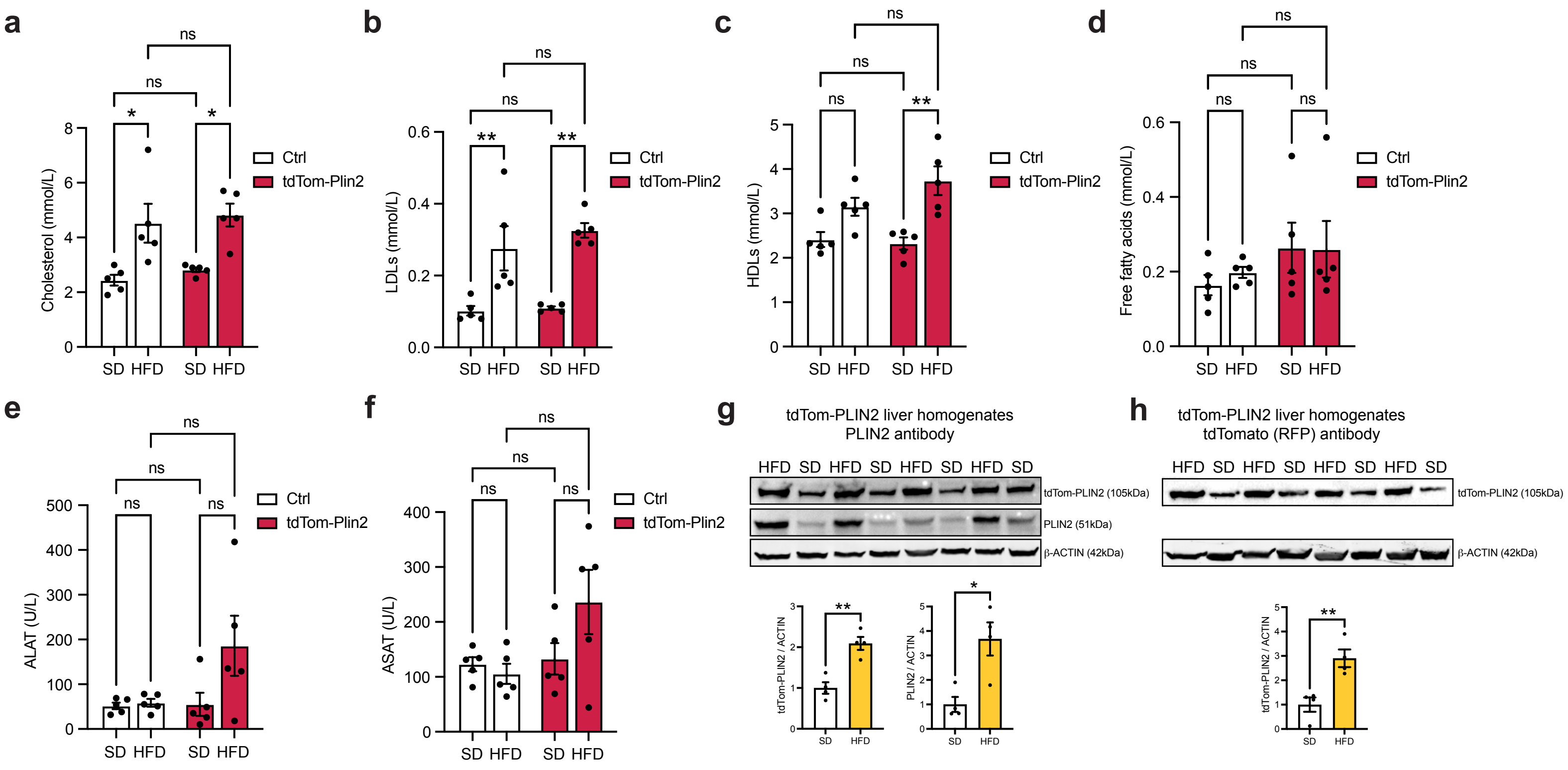

Supplementary Figure 2. TdTom-Plin2 mice increase fat mass and LDs in the liver upon a short-term high fat diet, related to Figure 2.

a, b and **c)** Serum analysis of Ctrl and tdTom-Plin2 mice on SD or HFD show a significant increase in cholesterol, low-density lipoprotein (LDL) and high-density lipoprotein (HDL) after HFD. (n=5 mice per group, mean +/- SEM). **d)** Free fatty acids in the serum were not significantly changed. **e** and **f**) Blood levels of alanine aminotransferase (ALAT) and aspartate aminotransferase (ASAT), which are used as indicators of liver disease, were not significantly changed with HFD, even though tdTom-Plin2 mice on HFD had a slight elevation in both. **g** and **h)** Western blot analysis of tdTom-Plin2 liver homogenates from SD and HFD fed mice. The increase in PLIN2 protein can be seen in both the tagged and untagged version, revealed by an antibody against PLIN2, or by detecting only the tagged version using an anti tdTomato (RFP) antibody (n=4 mice per condition, mean +SEM). Asterisks indicate the following p-values: *< 0.05. **< 0.01 ns= non-significant.

Supplementary Figure 3

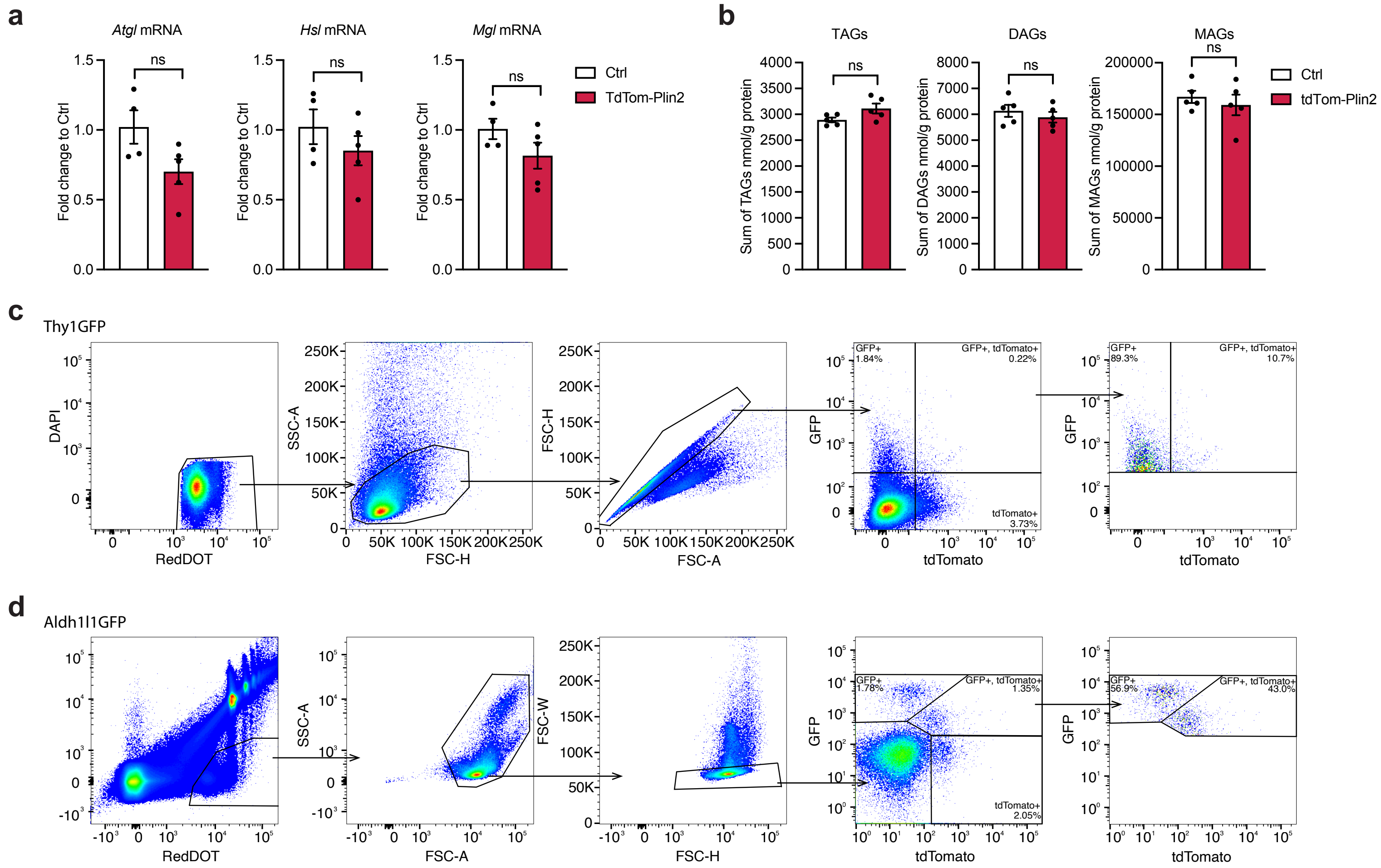

Supplementary Figure 3. LDs are present in multiple cell types in the healthy adult mouse brain, related to Figure 3.

a) Analysis of mRNA expression by RT-qPCR show no significant difference in the lipases *Atgl, Hsl* and Mgl in the brain of Ctrl and tdTom-Plin2 mice. (n=4 Ctrl and 5 tdTom-Plin2 male mice, measured in duplicates, fold change +/- SEM). **b)** There are no significant differences in brain TAGs, DAGs and MAGs, measured by lipidomics analysis, between Ctrl and tdTom-Plin2 mice. (n=5 mice per group, mean +/- SEM). **c** and **d)** FACS gating strategy for Thy1GFP positive neurons and Aldh1l1GFP positive astrocytes. All samples were first selected on viability based on DAPI and RedDOT staining, followed by sorting based on size and granularity, exclusion of doublets to have a population of viable single cells. These were then sorted based on GFP (cell marker) and tdTomato (tdTom-Plin2) to quantify how many cells have LDs in the different cell populations of the brain.

Supplementary Figure 4

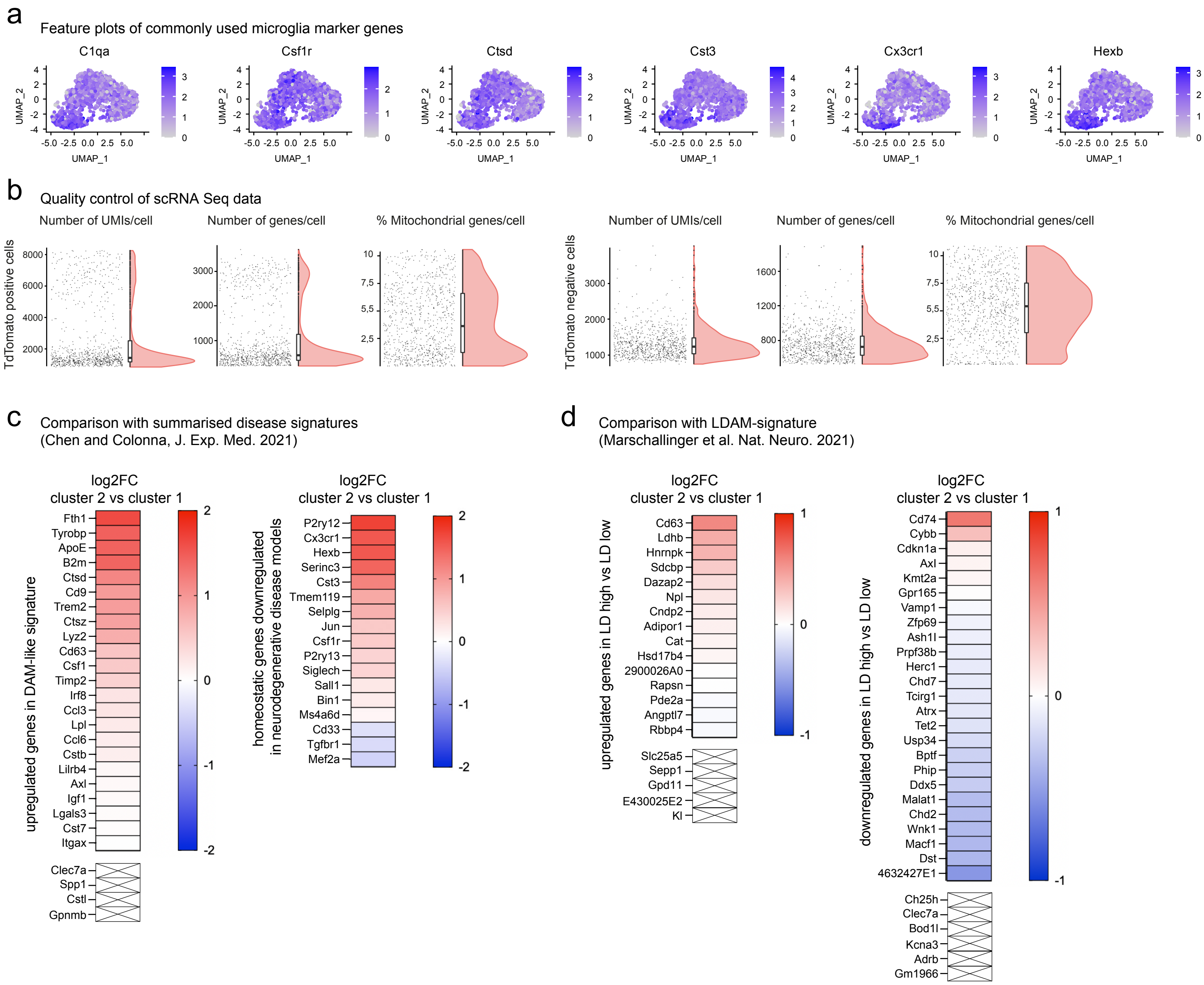

Supplementary Figure 4. ScRNA sequencing of tdTomato positive microglia reveals a subpopulation with a specific signature under physiological conditions, related to Figure 4.

a) Feature plots of commonly used microglia marker genes shows the microglial nature of the cells analysed. **b)** Quality control of the scRNA seq data, showing number of Unique Molecular Identifiers (UMIs), detected gene number, and mitochondrial gene percentage, indicative of good data quality for cells from adult mice. **c)** Comparison of the gene signature of the tdTom-Plin2 enriched cluster 2 with the summarised disease signatures published by Chen and Colonna (Chen and Colonna, 2021). **d)** Comparison of the gene signature of the tdTom-Plin2 enriched cluster 2 with the LDAM signature published by Marschallinger and colleagues (Marschallinger et al., 2020).

Supplementary Figure 5

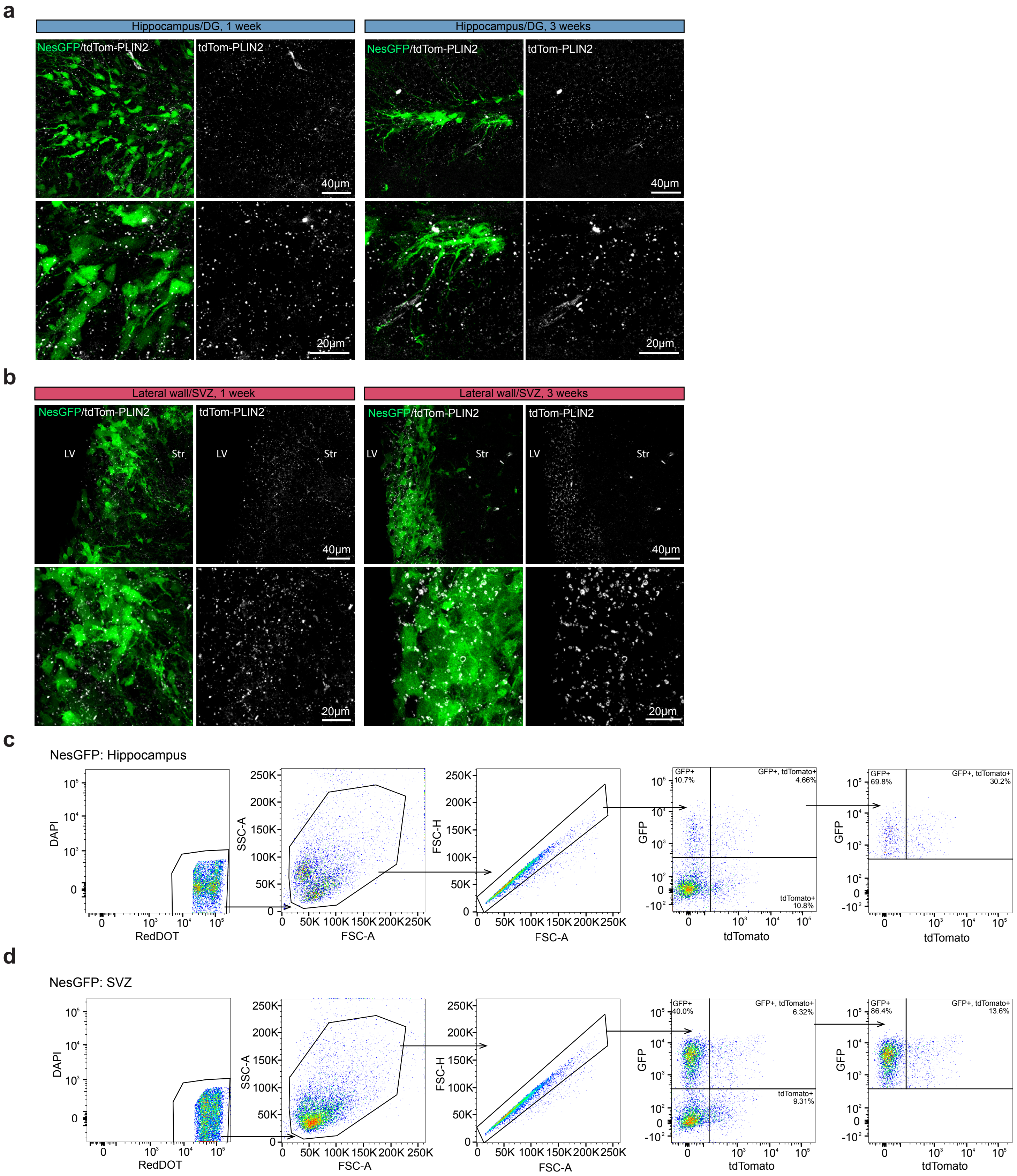

Supplementary Figure 5. LDs are present in NSPCs and their progeny in the postnatal and adult mouse brain, related to Figure 5.

a and **b)** tdTom-PLIN2 reveals that LDs are abundant the DG and SVZ of both 1 week and 3 week old mice. Representative images of non-stained sections showing NesGFP positive NSPCs and tdTom-PLIN2 positive LDs in the DG and SVZ. (maximum intensity projections, 20 μm stacks). **c** and **d)** FACS gating strategy for NesGFP positive cells in the hippocampus and SVZ. All samples were first selected on viability based in DAPI and RedDOT staining, followed by sorting based on size and granularity, exclusion of doublets to have a population of viable single cells. These were then sorted based on GFP (cell marker) and tdTomato (tdTom-PLIN2) to quantify how many cells have LDs.

Supplementary Figure 6

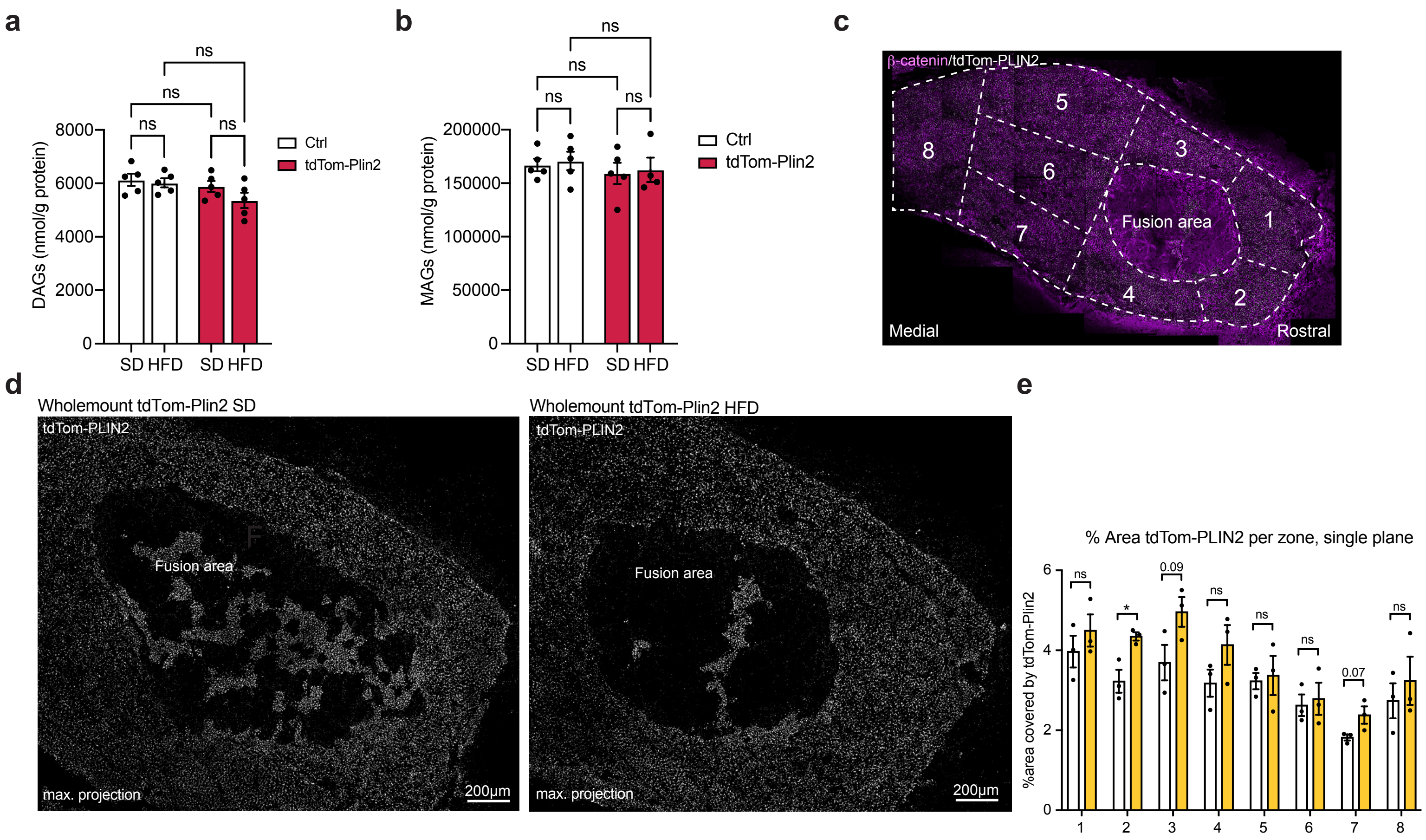

Supplementary Figure 6. Short-term high fat diet increases the total TAG levels in the brain and leads to slightly increased LDs in the wall of the lateral ventricles, related to Figure 6.

a and **b**) Lipidomic analysis show no significant difference in DAGs and MAGs after HFD in neither Ctrl or tdTom-Plin2 mice. (n=5 mice per group, mean +/- SEM, for MAGs, one HFD tdTom-Plin2 mouse was excluded as an outlier). **c)** Outline of the zones analysed in the wholemount preparations of SD and HFD tdTom-Plin2 mice. **d)** Wholemount preparation of the lateral ventricle showing LDs stored in ependymal cells and NSPCs in both SD (left panel) and HFD (right panel) mice. **e)** Quantification of the area covered by tdTom-Plin2 in the different zones. (n=3 mice per group, mean +/- SEM).

Asterisks indicate the following p-values: * < 0.05; ns= non-significant.

Supplementary Figure 7

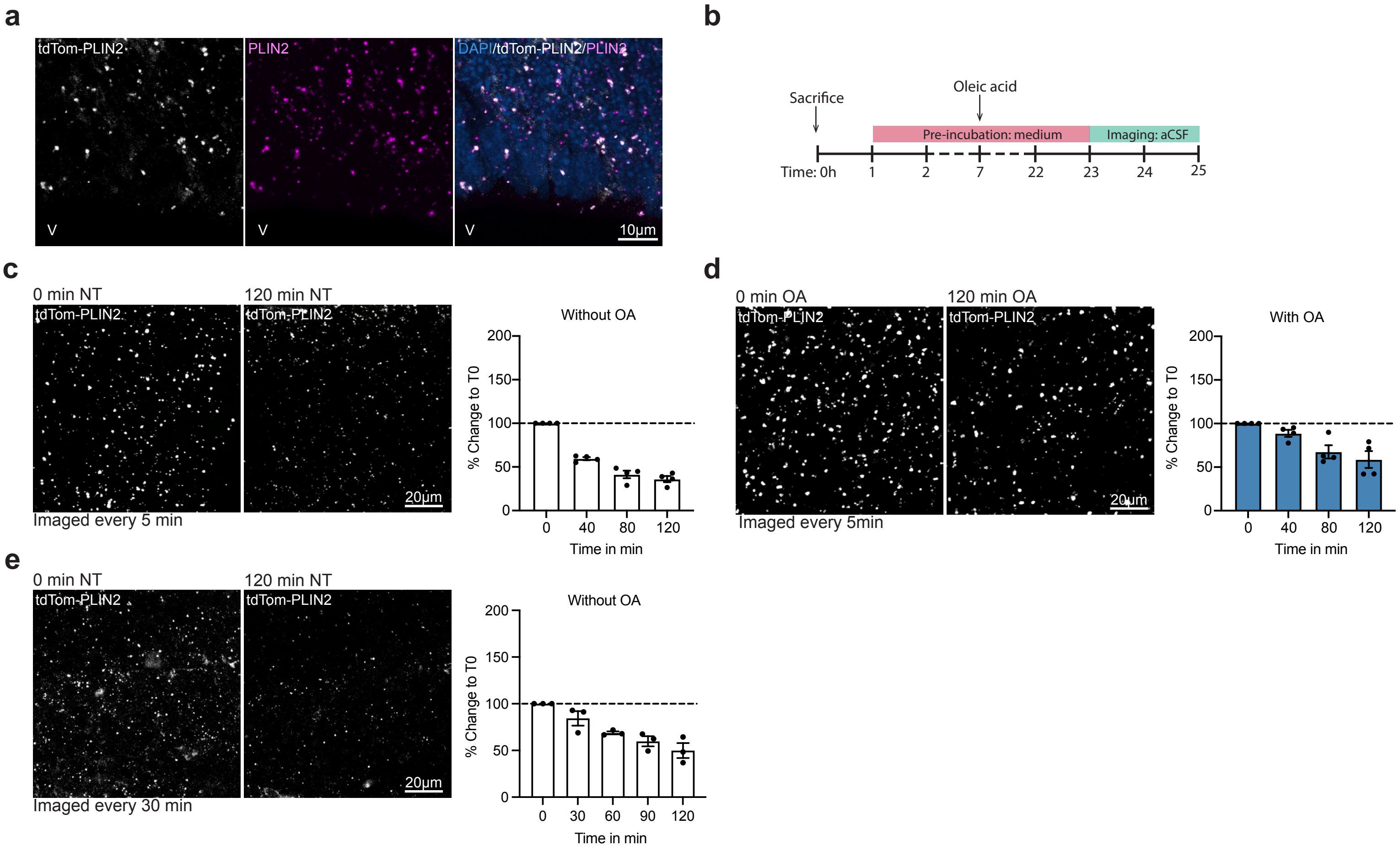

Supplementary Figure 7. LDs are present in the developing embryonic brain and react dynamically to exogenous lipids, related to Figure 7.

a) Immunohistochemical staining with PLIN2 shows good colocalization of PLIN2 with tdTom-Plin2. **b)** Timeline of OA treatment before live imaging. **c** and **d)** Representative images showing tdTom-Plin2-positive LDs in NT and OA treated sections at the start point (0 min) and endpoint (120 min) of the live imaging. Maximum intensity projections, 30 μm stacks. (n=4 embryos, mean +/- SEM). **e)** Repetition of the experiment shown in **c**, but imaged every 30 min to reduce a potential effect of photobleaching (n=3 embryos, mean +/- SEM).

Supplementary Figure 8

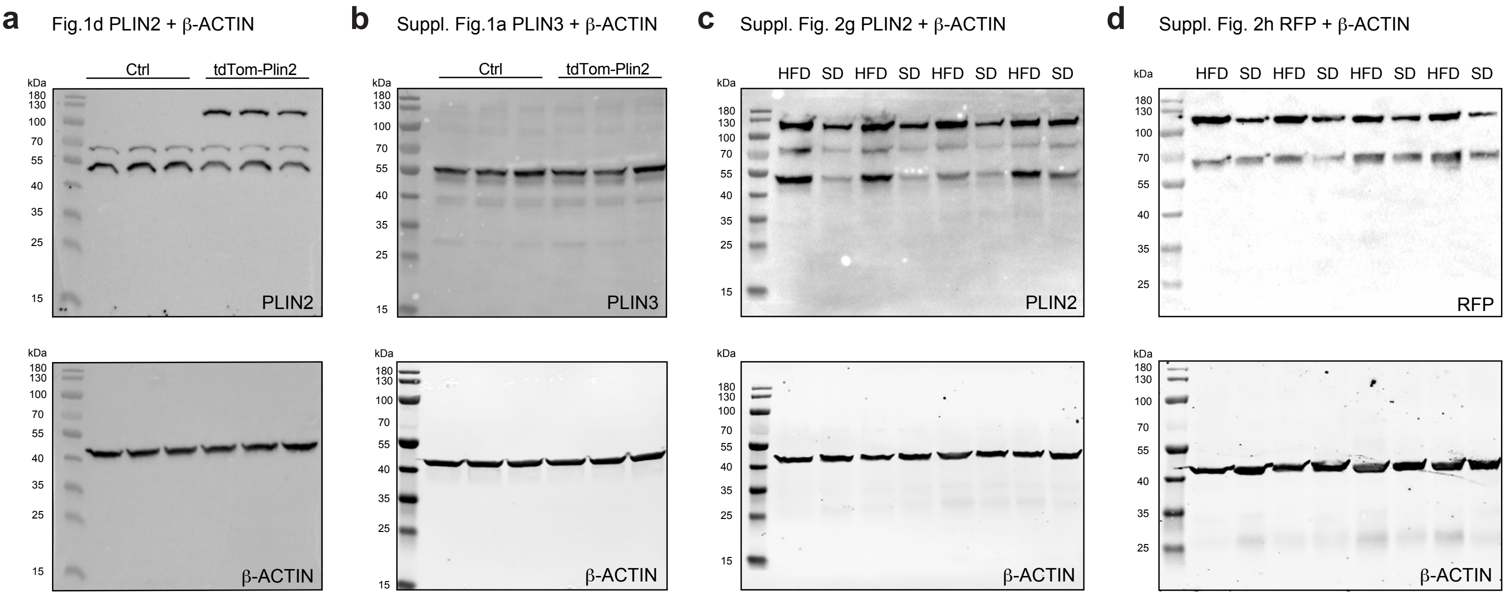

Supplementary Figure 8. Uncropped Western blots

Uncropped Western blots displayed in Figure 1D (**A**), Figure S1A (**B**), Figure S2G (**C**) and Figure S2H (**D**).
