# Supplementary material for: A fluorescent perilipin 2 knock-in mouse model reveals a high abundance of lipid droplets in the developing and adult brain
Source: Nat Commun. 2024 Jun 28;15:5489. doi: 10.1038/s41467-024-49449-w (PMC11213871; doi:10.1038/s41467-024-49449-w)
Supplement: Supplementary file 3 — Description of Additional Supplementary Files [file 41467_2024_49449_MOESM3_ESM.pdf]

Description of Additional Supplementary Files:

Supplementary Data 1: Proteomics data comparison tdTom-Plin2 wt and tdTom-Plin2 het NSPCs

Supplementary Data 2: Differentially expressed genes in microglia cluster 2 vs cluster 1
