# Supplementary material for: A fluorescent perilipin 2 knock-in mouse model reveals a high abundance of lipid droplets in the developing and adult brain
Source: Nat Commun. 2024 Jun 28;15:5489. doi: 10.1038/s41467-024-49449-w (PMC11213871; doi:10.1038/s41467-024-49449-w)
Supplement: Supplementary file 6 — Reporting Summary [file 41467_2024_49449_MOESM6_ESM.pdf]

nature portfolio

Corresponding author(s): Marlen Knobloch

Last updated by author(s): May 24, 2024

Reporting Summary

Nature Portfolio wishes to improve the reproducibility of the work that we publish. This form provides structure for consistency and transparency in reporting. For further information on Nature Portfolio policies, see our Editorial Policies and the Editorial Policy Checklist.

Statistics

For all statistical analyses, confirm that the following items are present in the figure legend, table legend, main text, or Methods section.

n/a	Confirmed	
☐	☒	The exact sample size (*n*) for each experimental group/condition, given as a discrete number and unit of measurement
☐	☒	A statement on whether measurements were taken from distinct samples or whether the same sample was measured repeatedly
☐	☒	The statistical test(s) used AND whether they are one- or two-sided<br>*Only common tests should be described solely by name; describe more complex techniques in the Methods section.*
☐	☒	A description of all covariates tested
☐	☒	A description of any assumptions or corrections, such as tests of normality and adjustment for multiple comparisons
☐	☒	A full description of the statistical parameters including central tendency (e.g. means) or other basic estimates (e.g. regression coefficient) AND variation (e.g. standard deviation) or associated estimates of uncertainty (e.g. confidence intervals)
☒	☐	For null hypothesis testing, the test statistic (e.g. *F*, *t*, *r*) with confidence intervals, effect sizes, degrees of freedom and *P* value noted<br>*Give P values as exact values whenever suitable.*
☒	☐	For Bayesian analysis, information on the choice of priors and Markov chain Monte Carlo settings
☒	☐	For hierarchical and complex designs, identification of the appropriate level for tests and full reporting of outcomes
☒	☐	Estimates of effect sizes (e.g. Cohen's *d*, Pearson's *r*), indicating how they were calculated

Our web collection on statistics for biologists contains articles on many of the points above.

Software and code

Policy information about availability of computer code

Data collection	Data collection, such as image acquisitions, metabolic measurements etc. were performed with the specific instrument softwares installed on the instruments, as detailed in the methods.
Data analysis	Data analysis was performed with the following softwares:<br>Image analysis: Fiji (Version 2.0.0-rc-69/1.52p), Imaris (Version 9.8, https://imaris.oxinst.com/).<br>scRNA seq data: Seurat 4.0.4 in R 3.6.1<br>Flow Cytometry: FlowJo 10.6.2.<br>Proteomics: Spectronaut 18.1.230626.50606 (Biognosys, Schlieren, Switzerland), Perseus software package (version 1.6.15.0)<br>Lipidomics: MultiQuant Software (version 3.0.3, Sciex technologies), R” software (http://cran.r-project.org/<br>Statistical analyses: Prism Graphpad (10.1.1)<br>Details of the software codes used are described in the method section and are available from the corresponding author on request

For manuscripts utilizing custom algorithms or software that are central to the research but not yet described in published literature, software must be made available to editors and reviewers. We strongly encourage code deposition in a community repository (e.g. GitHub). See the Nature Portfolio guidelines for submitting code & software for further information.

Data

Policy information about availability of data

All manuscripts must include a data availability statement. This statement should provide the following information, where applicable:

- Accession codes, unique identifiers, or web links for publicly available datasets
- A description of any restrictions on data availability
- For clinical datasets or third party data, please ensure that the statement adheres to our policy

The data supporting the findings from this study are available within the article file and its supplementary information. scRNA-seq raw and preprocessed data generated in this study have been deposited in the GEO database under accession code GSE267069. All raw MS data together with raw output tables are available via the Proteomexchange data repository (www.proteomexchange.org) with the accession PXD051943.
Any other raw data or noncommercial material used in this study are available from the corresponding author upon reasonable request. Details of the software codes used are described in the method section and are available from the corresponding author on request.
A source data file is provided with the paper.

Research involving human participants, their data, or biological material

Policy information about studies with human participants or human data. See also policy information about sex, gender (identity/presentation), and sexual orientation and race, ethnicity and racism.

Reporting on sex and gender	*Use the terms sex (biological attribute) and gender (shaped by social and cultural circumstances) carefully in order to avoid confusing both terms. Indicate if findings apply to only one sex or gender; describe whether sex and gender were considered in study design; whether sex and/or gender was determined based on self-reporting or assigned and methods used. Provide in the source data disaggregated sex and gender data, where this information has been collected, and if consent has been obtained for sharing of individual-level data; provide overall numbers in this Reporting Summary. Please state if this information has not been collected. Report sex- and gender-based analyses where performed, justify reasons for lack of sex- and gender-based analysis.*
Reporting on race, ethnicity, or other socially relevant groupings	*Please specify the socially constructed or socially relevant categorization variable(s) used in your manuscript and explain why they were used. Please note that such variables should not be used as proxies for other socially constructed/relevant variables (for example, race or ethnicity should not be used as a proxy for socioeconomic status). Provide clear definitions of the relevant terms used, how they were provided (by the participants/respondents, the researchers, or third parties), and the method(s) used to classify people into the different categories (e.g. self-report, census or administrative data, social media data, etc.) Please provide details about how you controlled for confounding variables in your analyses.*
Population characteristics	*Describe the covariate-relevant population characteristics of the human research participants (e.g. age, genotypic information, past and current diagnosis and treatment categories). If you filled out the behavioural & social sciences study design questions and have nothing to add here, write "See above."*
Recruitment	*Describe how participants were recruited. Outline any potential self-selection bias or other biases that may be present and how these are likely to impact results.*
Ethics oversight	*Identify the organization(s) that approved the study protocol.*

Note that full information on the approval of the study protocol must also be provided in the manuscript.

Field-specific reporting

Please select the one below that is the best fit for your research. If you are not sure, read the appropriate sections before making your selection.

☒ Life sciences ☐ Behavioural & social sciences ☐ Ecological, evolutionary & environmental sciences

For a reference copy of the document with all sections, see nature.com/documents/nr-reporting-summary-flat.pdf

Life sciences study design

All studies must disclose on these points even when the disclosure is negative.

Sample size	A minimum of n=3 samples was used for each analysis. The nature of the n is described for each experiment in the corresponding figure legends and all detailed values are provided in the Source Data file. Sample size determinations are based on previous experience and standards in the field.
Data exclusions	Data were analysed for outliers using the Grubbs outlier test (Graph pad Prism). In a few cases, outliers were excluded, as specified in the Source Data file.
Replication	Experiments were repeated as indicated in details for each figure panel and as described in the methods. The replications are shown as individual dots together with the calculated means and variability (standard errors of the means).

Randomization	For the HFD experiments, mice were randomly assigned to the diet within each genotype. For a subcohort, het and Ctrl littermates were assigned pairwise to the same diet.
Blinding	Data acquisition and/or analyses in these studies were performed in a blinded way.

Reporting for specific materials, systems and methods

We require information from authors about some types of materials, experimental systems and methods used in many studies. Here, indicate whether each material, system or method listed is relevant to your study. If you are not sure if a list item applies to your research, read the appropriate section before selecting a response.

Materials & experimental systems

n/a	Involved in the study
☐	☒ Antibodies
☐	☒ Eukaryotic cell lines
☒	☐ Palaeontology and archaeology
☐	☒ Animals and other organisms
☒	☐ Clinical data
☒	☐ Dual use research of concern
☒	☐ Plants

Methods

n/a	Involved in the study
☒	☐ ChIP-seq
☐	☒ Flow cytometry
☒	☐ MRI-based neuroimaging

Antibodies

Antibodies used	All antibodies used in this study are listed in the method section. Dilutions, provider and ordering numbers are given as well as the protocol used.
Validation	All antibodies used in this study are from commercial suppliers that have verified the specificity of the antibodies using recombinant proteins or knock-out lines. All the antibodies have been previously used by various laboratories. All secondary antibodies are verified to not give a specific staining without the primary antibody.

Eukaryotic cell lines

Policy information about cell lines and Sex and Gender in Research

Cell line source(s)	All NSPCs used in this study were derived from adult mice by ourselves. Cells were kept as "lines" for many passages (up to P25).
Authentication	not applicable
Mycoplasma contamination	All NSPCs used in this study were regularly tested for mycoplasma contamination and were negative.
Commonly misidentified lines (See ICLAC register)	not applicable

Animals and other research organisms

Policy information about studies involving animals; ARRIVE guidelines recommended for reporting animal research, and Sex and Gender in Research

Laboratory animals	The tdTom-Plin2 line was newly created as described in details in the method sections. Both males and females were used for the study. The age of the mice is indicated in each figure and ranged from Embryonic age E14.5 to adult mice (8 weeks of age). All mice were kept under standard conditions in ventilated cages with ad libitum food and water. Housing conditions were as following: dark/light cycle 12/12, ambient temperature around 21–22 °C and humidity between 40 and 70% (55% in average).
Wild animals	The study did not involve wild animals.
Reporting on sex	The data presented in the study are performed with male mice. Similar experiments were also performed with female mice and results were comparable. However, as there was a larger variability (for instance for the HFD experiments), more mice will need to be included for the experiments with the female mice.
Field-collected samples	no field-collected samples

Ethics oversight | All experiments including animals were carried out in compliance with the Swiss law after approval from the local authorities (Cantonal veterinary office, Canton de Vaud, Switzerland).

Note that full information on the approval of the study protocol must also be provided in the manuscript.

Plants

Seed stocks | *Report on the source of all seed stocks or other plant material used. If applicable, state the seed stock centre and catalogue number. If plant specimens were collected from the field, describe the collection location, date and sampling procedures.*

Novel plant genotypes | *Describe the methods by which all novel plant genotypes were produced. This includes those generated by transgenic approaches, gene editing, chemical/radiation-based mutagenesis and hybridization. For transgenic lines, describe the transformation method, the number of independent lines analyzed and the generation upon which experiments were performed. For gene-edited lines, describe the editor used, the endogenous sequence targeted for editing, the targeting guide RNA sequence (if applicable) and how the editor was applied.*

Authentication | *Describe any authentication procedures for each seed stock used or novel genotype generated. Describe any experiments used to assess the effect of a mutation and, where applicable, how potential secondary effects (e.g. second site T-DNA insertions, mosiacism, off-target gene editing) were examined.*

Flow Cytometry

Plots

Confirm that:

- [x] The axis labels state the marker and fluorochrome used (e.g. CD4-FITC).
- [x] The axis scales are clearly visible. Include numbers along axes only for bottom left plot of group (a 'group' is an analysis of identical markers).
- [x] All plots are contour plots with outliers or pseudocolor plots.
- [x] A numerical value for number of cells or percentage (with statistics) is provided.

Methodology

Sample preparation | Cells and single cell brain suspensions used for FACS were prepared as detailed in the method section. The cells were kept on ice and sorting was performed at 4°C.

Instrument | MoFlo Astrios EQcell sorter (Beckman Coulter, UNIL FACS facility), Cytoflex S Flow cytometer (Beckman Coulter)

Software | Flow Cytometry data were acquired with the instrument software and analyzed with FlowJo 10.6.2.

Cell population abundance | Cells were sorted in purity mode

Gating strategy | Gating strategies are shown for each experiment in the supplementary information. They were established using single transgenic mice where applicable. In general, the first gating is done on viable cells to exclude debris and dead cells, followed by the selection of single cells (excluding doublets or clumps). Only then was the specific fluorophore (such as for instance GFP and tdTomato) taken into account.

- [x] Tick this box to confirm that a figure exemplifying the gating strategy is provided in the Supplementary Information.
